# St. Louis Encephalitis Virus Infection in Woman, Peru

**DOI:** 10.3201/eid2004.131735

**Published:** 2014-04

**Authors:** Vidal Felices, Julia S. Ampuero, Carolina Guevara, Edna R. Caceda, Jorge Gomez, Felix W. Santiago-Maldonado, Patricia V. Aguilar, Eric S. Halsey

**Affiliations:** US Naval Medical Research Unit No. 6, Lima, Peru (V. Felices, J. Ampuero, C. Guevara, E. Caceda, E. Halsey);; Dirección General de Epidemiología-Ministerio de Salud, Lima (J. Gomez);; University of Texas Medical Branch, Galveston, Texas, USA (F. Santiago-Maldonado, P. Aguilar)

**Keywords:** flavivirus, flavivirus infections, arbovirus, arbovirus infections, pharyngitis, respiratory signs and symptoms, South America, viruses, vector-borne diseases, Peru

**To the Edito**r: St. Louis encephalitis virus (SLEV) is a flavivirus that can asymptomatically infect humans or cause clinically apparent disease that manifests with fever, headache, nausea, and vomiting (*1*). More severe disease with meningoencephalitic involvement may result in stiff neck, alteration in consciousness, gait disturbance, and other focal neurologic deficits. Heightened levels of human disease are often associated with increased abundance of *Culex* spp. mosquitoes and the summer season.

SLEV was first reported in South America in 1960, when it was isolated from pools of *Sabethes bellisarioi* mosquitoes and *Gigantolaelaps* mites in Pará, Brazil (*2*). SLEV was later recovered from humans in Argentina (1963) (*3*) and Brazil (1978) (*4*). Sporadic infections and large outbreaks occurred over ensuing decades, although no isolates in humans have been reported in other South American countries.

Serologic indication of SLEV circulation in Peru was first obtained from hemagglutination-inhibition and neutralization tests of samples collected in 1965 from residents of eastern Peru (*5*). Later, SLEV was isolated from mosquitoes (*6**,**7*), and SLEV antibody was detected in serum specimens from humans by plaque reduction neutralization tests (*6*). We report the isolation of SLEV from a person in Peru and describe a unique collection method, using oropharyngeal swab specimens, for detecting this virus.

In March 2006, a 50-year-old woman with a 1-day history of fever, sore throat, cough, malaise, myalgia, and headache sought treatment at her local health center in Quistococha, Peru, in the Amazon Basin (3°49′40′′ N; 73°19′6′′ E). The woman’s recent travel was limited to a 70-km radius from this town. Because influenza was suspected, an oropharyngeal swab specimen was collected as part of an influenza-like illness surveillance project, which had been approved by the US Naval Medical Research Center Institutional Review Board and endorsed by the Peruvian Ministry of Health. No blood specimen was obtained because blood was not collected in this respiratory infection–focused protocol. The swab specimen was inoculated onto Madin-Darby canine kidney cells; no cytopathic effect was observed, and the culture was negative for influenza virus and for other respiratory viruses amenable to culture (e.g., adenovirus and parainfluenza virus).

Nearly 6 years later, as part of a retrospective study of previously negative respiratory specimens, the sample was reevaluated for arboviral infection. Universal transport medium (Copan Diagnostics Inc., Murrieta, CA, USA), containing the swab specimen, was inoculated onto Vero 76 cells; cytopathic effects were revealed on day 7. The cells were harvested, and an indirect immunofluorescence assay was performed by using a panel of mouse polyclonal hyperimmune ascitic fluid specific to a variety of flaviviruses. The initial screening tests indicated reactivity to yellow fever virus, dengue virus, and SLEV. Subsequent immunofluorescence assay analyses using monoclonal antibodies against yellow fever virus and all 4 dengue virus serotypes were negative.

Viral RNA was recovered from the Vero culture supernatant and amplified by conventional reverse transcription PCR/nested PCR with generic flavivirus primers against the nonstructural 5 coding region, which confirmed that the isolate was a flavivirus. Real-time reverse transcription PCR with specific SLEV primers confirmed SLEV.

A total of 10,850 bp, almost the full genome sequence of the virus, were sequenced (GenBank accession no. KF589299), and 10,236 nt from these sequences were compared with other SLEV sequences in GenBank. The strain showed 98.4% similarity with a SLEV strain isolated from a bird in Brazil in 1973, 98.4% similarity with an SLEV strain isolated from mosquitoes in Peru in 1975, and 97.9% similarity with an SLEV strain isolated from mosquitoes in the United States in 2003. The US strain may have been carried by migratory birds from Latin America (*8*). Phylogenetic analysis by the neighbor-joining method with 1,000 bootstraps replicates identified the isolate as genotype V, subgenotype A, which grouped with the strains obtained in Brazil, Peru, and the United States (Figure) and with an SLEV genotype V strain obtained in Trinidad.

**Figure:**
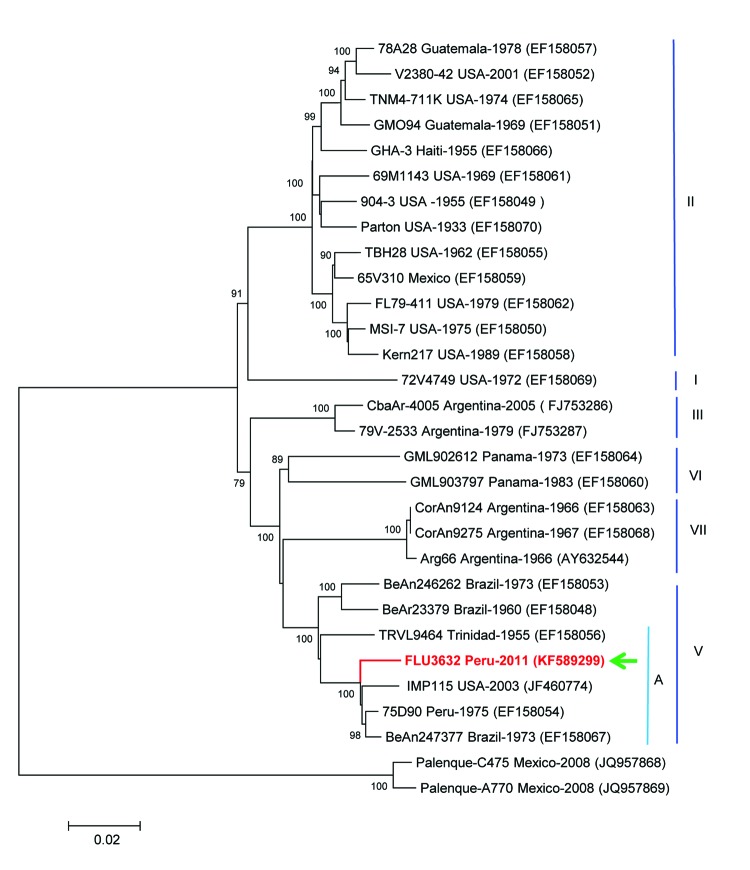
Phylogenetic analysis from the initially sequenced 10,850-nt region of the St. Louis encephalitis virus (SLEV) genome, isolated from a woman in Peru, 2006. The sequence possessed only 92.8% homology with the NS5 gene region of the sole preexisting SLEV in the laboratory, a genotype II strain similar to TBH28 USA. The Peruvian SLEV sequence described in this case (FLU3632, arrow) groups with Brazil (1975), Peru (1973), and USA (2003) strains, inside the genotype V, subgenotype A. The evolutionary history was inferred by using the neighbor-joining method based on the Kimura 2-parameter model. The entire open reading frame sequence of the Peruvian SLEV isolate was determined in this study by using the Illumina HiSeq 1000 system (Illumina, Inc., San Diego, CA, USA) and assigned GenBank accession no. KF589299. Sequences were analyzed and assembled using the SeqMan Lasergene V.5 and then compared with sequences deposited in GenBank. Multiple sequence alignments were performed by using ClustalX version 2.0.10 (Conway Institute, University College, Dublin, Ireland; www.clustal.org/) and BioEdit version 7.0.9.0. (Ibis Biosciences, Carlsbad, CA, USA) Genetic divergence was determined by using MEGA version 5.02 (www.megasoftware.net/). Scale bar indicates nucleotide substitutions per site.

Our findings bolster previous serologic investigations, adding Peru to the South American countries reporting this virus in humans. This discovery is not surprising because *Culex* spp. mosquitoes, the main vectors of SLEV in Brazil and Argentina, have been shown to carry SLEV in Peru (*1**,**7*).

Although SLEV disease in humans is usually confirmed by testing blood or cerebrospinal fluid, the isolate described here was recovered from an oropharyngeal swab specimen. The presence of this virus in human respiratory samples was previously suggested by a study in which nasal wash samples from SLEV-infected persons were injected intranasally into mice, which induced immunity to a subsequent intracerebral challenge with SLEV (*9*). Although this circumstance is uncommon, other arboviruses have also been obtained from the upper respiratory tract, including dengue virus from nasal and throat swab specimens (*10*).

Our findings have many implications. First, SLEV may cause human disease in a wider area of South America than was previously known. In addition, SLEV can cause influenza-like illness and may elude identification unless specific assays are used. Finally, the upper respiratory tract may offer a less invasive way of recovering SLEV isolates than by lumbar puncture or even by drawing blood, although further investigations are needed.
